# The Abortion of Smallpox—An Important Secret

**Published:** 1881-04

**Authors:** 


The Abortion of Smallpox—An Important Secret.—Not long since a case of sm llpox was reported to the Board of Health of one of our California towns by an individual holding a homœopathic license and advertising himself as "a physician of experience." Inspection by the Health Officer proved that it was a case of chronic cutaneous disease having no relation to smallpox. The "doctor" explained that his diagnosis was correct, but that he had cut the disease short by his treatment (homœopathic) and that it did not develop. The affair was taken up by the people and by the newspapers, and the discussion of it brought out the following communication, which deserves perblicity, not only on account of the invaluable process to which it refers, viz., cutting short completely a terrible disease, but by reason of its literary merit. It is copied from the original with scrupulous exactness.

I have just red the article in the ——— of January 27 Headed erroneous diagnosis. under Letters from the People He admits the errer in the start. The card of explanation has been dessected and reconstructed. I said papules, vesicles and pustules. Non of that nonsense. The treatment did so mitigate the disease that it was not recognized as smallpox, but "Ring-Worm." The case can speak for itself, for it was not a failure, I stand corrected in punctuation, When I wrote that letter it was in haste in order to leave that office. I did treat a case of varioloid there. The Young Lady did not want to go to the pest house. The assertion's are not correct. she was a strainger had no friends. These case's wer semalar. both don well. I did not have to berrey them as failure's. Did not pass the board of examener's as stated that is fals. my Diploma's Hospital certificates, and Cards of the different colleges can be seen at my office at any time. The rest I ignore.—*Pacific Medical and Surical Journal.* * * * M D.

